# Diversity and relationships among strains of culturable yeasts in agricultural soils in Cameroon

**DOI:** 10.1038/s41598-018-34122-2

**Published:** 2018-10-24

**Authors:** Renad Aljohani, Himeshi Samarasinghe, Tabi Ashu, Jianping Xu

**Affiliations:** Department of Biology, McMaster University, 1280 Main St West, Hamilton, Ontario L8S 4K1 Canada

## Abstract

Yeasts are unicellular fungi; they are found in a diverse range of natural habitats, including soil, aquatic environments, the surface of plants, and the skin and mucosal surfaces of animal hosts. A variety of yeasts have been found in the soil environment. However, most studies of soil yeasts have come from developed countries, and there is a dearth of research on soil yeasts in Africa. In this study, we analyzed 493 soil samples from nine geographical locations in Cameroon for yeasts, using a culture - based method. A total of 110 yeast isolates were obtained. Based on their sequences at the fungal barcode locus, the Internal Transcribed Spacer (ITS) regions of the nuclear ribosomal RNA gene cluster, the 110 yeast isolates were putatively identified as belonging to 16 yeast species, including 15 Ascomycetes and one Basidiomycete. Differences in yeast species distribution were observed among the analyzed geographic regions. PCR fingerprinting analyses identified a large number of genotypes among strains within each of the obtained yeast species. Significantly, there was little evidence of geographic clustering among yeast strains from any of the yeast species. Our results suggest that Cameroon contains significant yeast diversity and that gene flow is common among local and regional soil yeast populations.

## Introduction

The “Fungal Kingdom” comprises diverse groups of organisms that can be separated to different categories based on different sets of criteria. For example, based on their nutrient and energy sources, fungi are often classified into saprophytes, symbionts, and parasites. Saprophytic fungi obtain their nutrients from dead organic matter and they play crucial roles in nutrient cycling in natural ecosystems. Symbiotic fungi obtain their organic nutrients from live symbiotic partners such as plants while they also contribute to the survival and reproduction of their partners. In contrast, parasitic fungi such as pathogens of plants and animals obtain their nutrients from their interacting partner and in the process, cause deleterious effects on their partners’ survival and reproduction. Morphologically, fungi in their vegetative state can exist either unicellularly such as yeasts or multicellularly such as filamentous hyphae and macroscopic mushrooms. Though useful for descriptive purposes, neither the nutrient source-based nor the morphology-based groupings represent the true evolutionary relationships among fungi. For example, yeasts can be found in diverse fungal phyla such as in Ascomycota and Basidiomycota, the two major phyla in fungi^1^. Most yeasts are saprophytes while some are pathogens that can cause deadly diseases in humans and other animals. Ecologically, yeasts have been found in a diverse range of habitats, including soil, aquatic environments, plant surfaces, foods, and skin and mucosal surfaces of animal hosts. Due to the remarkable heterogeneity of soil across diverse geographic and climate zones, the soil environments represent a major ecological niche for different types of fungi, including yeast^2^. However, the genetic relationships among yeasts from soil and other environmental sources remain poorly understood.

Among the currently known yeasts, the Brewer’s/Baker’s yeast *Saccharomyces cerevisiae* is probably the best-known. This yeast has been the model eukaryotic organism for genetics and molecular biological research, and more recently for ecological and evolutionary investigations. Industrially, *Saccharomyces cerevisiae* has been used in the food and alcoholic beverages industries for thousands of years^3,4^. Recent population genetic and molecular ecological studies have revealed a large genetic diversity within *S. cerevisiae*, and with robust evidence for population structuring at intercontinental scales^5,6^. At local levels, evidence for geographic and ecological structuring of *S. cerevisiae* has also been reported. For example, in Austria, while a number of genotypes were broadly distributed across regions, unique genotypes were found within most individual locations. A recent study using whole-genome sequence data showed evidence of gene flow between populations of *S. cerevisiae* inhabiting vineyards and oak trees^7^. Together, these results suggest evidence of gene flow among both geographic and ecological populations of *S. cerevisiae*^5–7^. However, due to the close association between *S. cerevisiae* and humans in food, fermentation and scientific research, it’s been difficult to determine how much of the observed relationships among populations of this yeast was due to anthropogenic activities and how much was due to natural forces.

Similar to *S. cerevisiae*, many other yeast species are also of environmental, economic, and/or medical significance. They play important roles in nutrient cycling and can help release nutrients for crops in agricultural fields^8,9^. Industrially, there have been significant efforts to isolate and study environmental yeast populations as a repository of strains to help improve traditional strains^10^. On the other hand, some environmental yeasts are opportunistic pathogens of humans, and they pose serious threats to human health. However, how those yeast populations are structured in natural environments remain poorly understood. In addition, most studies of environmental yeasts have come from developed countries. In contrast, there is a dearth of research on yeasts from developing countries, especially those from Africa.

Both culture-based and culture-independent methods have been employed to study microbial diversity in nature, including yeast diversity. In culture-based studies, artificial media and incubation conditions that favor yeast growth while limiting other microbes are typically used to enrich the yeast population, followed by isolation and purification. These yeasts are then identified based on their morphological, physiological, and/or molecular characteristics. In contrast, the culture-independent method directly analyzes the community DNA by means of extracting all DNA from the sample (e.g., soil), amplifying specific target DNA fragments using PCR, sequencing the amplified fragments, and comparing the sequences with those of reference strains in databases. In both approaches, where DNA sequencing is concerned, the target locus is often the Internal Transcribed Spacer (ITS) regions within the nuclear ribosomal RNA gene cluster. The ITS is chosen because this locus is highly conserved within most fungal species and there is an extensive collection of publicly available fungal ITS sequences from which to compare the experimental data^11^. Each of the two approaches (culture-based and culture-independent) has its advantages and disadvantages. For instance, the culture-independent method can reveal difficult-to-culture and unculturable microbes that the culture-based method would miss. Indeed, a recent global meta-genomics study on natural fungal communities in soil found that a large number of soil fungal/yeast species remain unidentified^12^. In contrast, the culture-based method allows researchers to obtain pure strains that can be further studied to address other biological questions related to their phenotypic and genotypic characteristics.

At present, there is no culture-independent approach that only targets environmental yeasts. The main reason is that yeasts are phylogenetically broadly distributed and intermixed with filamentous fungi. As a result, it’s difficult to design PCR primers and molecular probes that will allow the analyses of all yeasts but no filamentous fungi. Instead, culture-independent studies of yeast diversity have been exclusively embedded in fungal diversity studies^11,12^. In this study, we employed the culture-based approach to isolate and analyze yeasts from 493 soil samples obtained from nine geographical locations in three provinces in Cameroon. Cameroon is a Central African nation along the Atlantic Ocean and is bordered by several countries, including Nigeria, Chad, Gabon, and Republic of the Congo. Cameroon has all the major climates and vegetation zones of the African continent and hence is known as “Africa in Miniature”. Specifically, Cameroon has high mountains in the southwest, a desert in the far north, and rain forests in the north central regions. With the African continent remaining as one of the least explored regions in terms of fungal diversity, we sought to characterize the diversity of yeasts in Cameroonian soil through culturing methods followed by sequencing of the ITS locus for species identification. While the culture-based method may not reveal all of the yeasts existing in our soil samples, the obtained yeasts can provide materials for more in-depth investigations on the genotypic diversities and geographic relationships among yeast strains and populations within and among species. In this study, the soil samples were obtained from two climatic regions in Cameroon, namely the “Monsoon” climate and the “Tropical Savanna” climate. Our analyses revealed the presence of a variety of yeast species in Cameroon, with evidence of abundant intra-specific genetic diversity and frequent gene flow among geographic and climatic populations of these yeasts.

## Material and Methods

### Sampling sites and sample collection

A total 493 soil samples were obtained from nine different geographical locations in three Cameroonian regions/provinces (Table 1), including four sites (Mbingo, Bambui, Njinkejum, and Babanki) in the Northwest (NW) Region, one site (Makepe) in the Littoral (LI) Region, and four sites (Eloundem, Mbalgong, Simbock, and Mbandoumou) in the Centre (CE) Region. Sampling was conducted in June 2016; the warmest month in Cameroon and with the highest rainfall in the Northwest Region. At each location, the top layer of agricultural soils was collected (within the top 5 cm, at approximately 1 gram of fine soil particles per sample. The sampled fields had been mainly used to grow corn over the past decade. Individual samples were at least 5 meters apart from each other in all four cardinal directions. The general topography and vegetation in each region have been described previously^13,14^. Once all the soil samples were collected, they were shipped to McMaster University in Canada, stored at 4 °C, and processed within 2 weeks after receiving. The soil pH was determined by adding 0.1 g of soil to 1 ml sterilized water, allowing the soil to rehydrate for at least 1 h at 23 °C, and then measuring the pH using a bench-top Orion pH meter.

**Table 1:** Sampling locations, sample sizes, and the yeast species diversity among soil samples from Cameroon.

Region	Local site	Geographic coordinates	Total no. of soil samples	Number of soil sample with yeasts	PH of local sites	Number of yeast species	Percent samples containing yeast	Yeast species diversity
Northwest	Mbingo	6.1640546–164054	51	29	6.5	6	56.8	0.78
Northwest	Bambui	6.046586–10.232775	50	14	6.3	8	28	0.9
Northwest	Njinikejum	6.254341–10.298138	50	19	6.4	6	38	0.6
Northwest	Babanki	6.121895–10.266294	49	11	6.9	5	22.4	0.7
Littoral	Makepe	4.146774–9.839220	47	25	7.01	6	53.19	0.81
Centre	Eloundem	5.403848–11.844950	98	11	6.97	4	11.2	0.48
Centre	Mbalgong	4.447906–11.902442	51	1	7.07	1	1.9	NA*
Centre	Simbock	4.639568–12.045264	52	0	7.10	0	0	NA*
Centre	Mbandoumou	3.779870–11.511865	45	0	7.02	0	0	NA*
Total	—	—	493	110	—	—	22.3	—
Mean ± SD	—	—			—	—	23.4 ± 22.2	—

Due to their small sample sizes, these samples were excluded from the Simpson’s Diversity Index calculations.

### Isolation of yeast

Yeast isolation followed a protocol established previously^15^. Briefly, for each soil sample, we first homogenized the soil particles by vortexing and then transferred ~0.2 g of the soil into 1 ml of sterilized Sabouraud Dextrose Broth (SDB) containing 0.035 mg/ml of the antibiotic chloramphenicol. The samples were then incubated for 48 hours at 30 °C. After incubation, the samples were streaked onto Yeast Extract-Peptone-Dextrose agar (YEPD) medium and further incubated for 48 hours at 30 °C. For each sample, a representative of each morphologically distinct yeast or yeast-like colony was sub-cultured onto YEPD agar and incubated for another 48 hours at 30 °C. The purified fresh yeast cells were then harvested for DNA extraction. A portion of the fresh yeast cells of each isolate was stored in 30% glycerol at −80 °C. For soil samples that failed to yield yeasts in the first attempt, additional attempts were made with three soil samples of 0.1 g each in 1 ml of SDB containing 0.035 mg/ml of the antibiotic chloramphenicol and incubated them at 25 °C, 30 °C, and 37 °C for 5–7 days respectively. After incubation, the samples were then streaked onto YEPD medium and further incubated for 48 hours at 30 °C for evidence of yeasts. Further purification and storage of yeasts followed that described in the first attempt.

### Yeast species identification

To identify the yeast species, we first extracted genomic DNA following the protocol described previously^16^. These DNA samples were then diluted to 10 ng/ml and used as templates for PCR amplification of the ITS region using primers ITS1 (5′ TCCGTAGGTGAACCTGCGG 3′) and ITS4 (5′ TCCTCCGCTTATTGATATGC 3′). The PCR protocol included an initial denaturation step of 95 °C for 5 minutes, followed by 45 cycles of 30 s at 95 °C, 30 s at 55 °C, and 30 s at 72 °C, with a final extension of 7 minutes^15,16^. The ITS PCR products were checked via electrophoresis on 1% agarose gel with 1x TAE buffer for 60 minutes at 125 V. The gels were stained with ethidium bromide, photographed under ultraviolet light, and the respective PCR products were then sequenced from both directions. Sequencing was performed at the MOBIX Laboratory of McMaster University. The ITS sequences for our isolates have been deposited into the GenBank under the following accession numbers: MG817527-MG817636.

The obtained ITS sequence from each isolate was compared to two databases, GenBank and UNITE, for yeast species identifications using the BLAST algorithm. Here, following the commonly used criteria^11,12^, an isolate with ITS sequence greater than 97% sequence identity to the closest known yeast species was taken as an affirmative species identification. In contrast, those showing less than 97% sequence identity to any known species in the database are tentatively designated as new species and assigned to the closest species group or genus. We would like to note that even though the 97% sequence identity at the fungal ITS locus is commonly used in metagenomics studies to separate sequences into different species, it is also well-known that a substantial number of known sister species pairs of yeasts show greater than 97% sequence identity^11,12^. In the case that our strain shows similar and very close relationships (>97% sequence identity) to two or more such known yeast species, the degree of sequence divergence between the known species pairs is used as a guide to determine the taxonomic affiliation of our strain, including whether our strain represents a potential new species.

### PCR fingerprinting

To investigate the potential genetic variation among isolates within individual yeast species that we obtained in this study, a PCR fingerprinting method was performed using two separate primers: (1) the M13-core sequence (5′GTAAAACGACGGCCAGT-3′), and (2) the repeat sequence (GACA)_4_ (5′GACAGACAGACAGACA-3′). These two primers have been commonly utilized to help differentiate strains within individual fungal species^15,17–19^. For each PCR fingerprinting reaction, a total volume of 10 µl containing 5 µl of 2x GO Taq Master mix (Promega), 2 µl of H_2_O, 1 µl of 10 µM primer and 2 µl of template DNA was used. For both primers, a PCR included a denaturing step at 98 °C for 2 min, followed by 45 cycles at 93 °C for 20 s, 50 °C for 45 s, 72 °C for 20 s, and then followed by a final extension step at 72 °C for 6 min. The PCR products were electrophoresed on 1% agarose gel for 2.5 hours at 80 V, and all bands were scored manually using the 100 bp and 1 kb ladders as references. The MEGA 7 software (version 7.0.25) was used to calculate the genetic relationships among yeast isolates based on the PCR fingerprint patterns^20^.

### Statistical analysis

The yeast isolation rates for soil samples from each of the nine locations and three regions were calculated. For each location/region, the relative abundance of each yeast species was determined by dividing the number of isolates within each species by the total number of the yeasts isolated in the locations/regions. The Simpson’s species diversity index was calculated using the formula as $(1-\sum {{\rm{p}}}_{{\rm{i}}}^{2})\,{\rm{N}}/({\rm{N}}-{\rm{1}})$; where p_i_ is the frequency of the *i*th species and N is the total number of yeast isolates. The diversity index value ranges from 0 to 1. The species diversity value of 0 means all isolates from a given site belong to the same specie; while the value of 1 means that every isolate in the population sample belongs to a different species.

Similarly, within each species, the genotypic diversity index was calculated using the same formula except that p_i_ represents the frequency of the *i*th genotype in the species and N is the total number of yeast isolates in that species. A genotypic diversity value of 0 means all isolates from the same species have the same genotype, while a value of 1 means a high genotype diversity where every isolate has a different genotype.

The statistical significance of yeast isolation rate differences and of species diversity differences between the locations were determined using a Chi-square test against the null hypothesis that there was no difference between different locations/regions. A Pearson correlation test was preformed between the isolation rate of yeasts obtained in this study and the isolation rate of *Aspergillus fumigatus* in the same locations^21^ using Excel (software XLSTAT 365, 2017).

## Results

### Yeast isolation and identification

**Figure 1:**
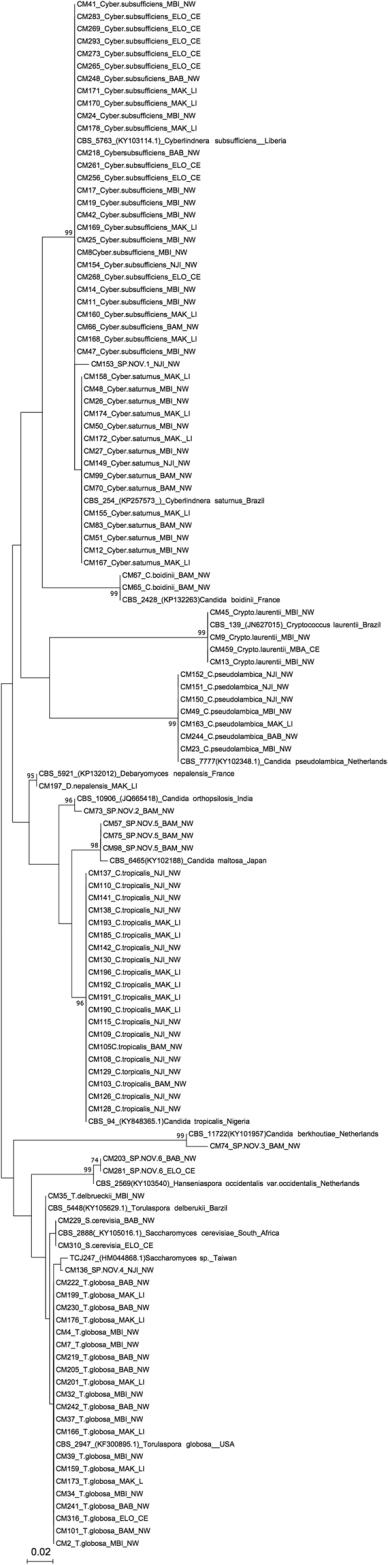
Phylogenetic relationships among the 110 isolates based on their ITS sequences. Their closest GenBank matches based on NCBI Blast and UNITE database searches are shown. For each strain, the soil sample identification number, the putative species that the strain belongs to, and the local and regional site where the soil sample was collected were shown. The location abbreviations as follows: (i) Mbingo (MBI), Bambui (BAM), Njinkejum (NJI), and Babanki (BAB) in the Northwest (NW) region; (ii) Makepe (MAK) in the Littoral (LI) region; and (iii) Eloundem (ELO), Mbalgong (MBAL), Simbock (SIM), and Mbandoumou (MBAN) in the Centre (CE) region.

A total of 110 yeast isolates were obtained from the 493 soil samples collected from nine geographical locations in Cameroon (Table 1). According to their ITS sequences (accession numbers MG817527-MG817636), the 110 isolates likely belonged to 16 different yeast species. Out of these 16 species, one was a basidiomycete (*Cryptococcus laurentii*), and the remaining 15 were ascomycetes. Nine of the 15 ascomycetes species belonged to known taxa: *Candida boidinii*, *Candida pseudolambica, Candida tropicalis, Cyberlindnera saturnus, Cyberlindnera subsufficiens, Debaryomyces nepalensis, Saccharomyces cerevisiae, Torulaspora delbrueckii*, and *Torulaspora globosa*. The remaining six species were putative new species as they either showed greater than 3% sequence divergence at the ITS locus from the six closest known yeast species or showed similar or greater divergence from the known species than their most closely related known sister species pairs. Among these six putative new species, two showed 98% and 99% ITS sequence identity to two sequences deposited in GenBank, but those two sequences were not yet associated with any species name. Here, these six putative new species are tentatively called novel species #1–6; they consisted of nine of the 110 isolates. Novel species 1 (SP.NOV.1) belonged to the ascomycete genus *Cyberlindnera*, while novel species 2 (SP.NOV.2), 3 (SP.NOV.3), and 5 (SP.NOV.5) were identified as most closely related to species in the genus *Candida*. Novel species 6 (SP.NOV.6) belonged to genus *Hanseniaspora*, while novel species 4 (SP.NOV.4) was found to be most closely related to organisms in the genus *Saccharomyces* (Table 2 and Fig. 1). Table 2 shows the accession numbers of the most closely related reference ITS sequences in the NCBI GenBank used to identify our yeast isolates, as well as the percentage of sequence identity. Among these 16 species or putative species, eight were found in more than one location, while the remaining eight species were each found in only one location (Tables 2 and 3).

**Table 2:** Summary information about the yeast species identified from Cameroon soil. Included in the table are the GenBank accession numbers of the closest reference strains, the sites where each species was found in our samples, the percentage of ITS sequence identity, and the number of yeast isolates in each species in our total Cameroonian sample.

Species	Closest GenBank Number	Sites	Percent sequence identity (%)	Number of isolates
*Candida boidinii*	KP132263	Bambui(NW)	99%	2
*Candida pseudolambica*	KM384611	Mbingo(NW) Njinikejum(NW) Makepe(LI) Babanki(NW)	98%	7
*Candida tropicalis*	KY102481	Bambui(NW) Njinikejum(NW) Makepe(LI)	99%	20
*Cryptococcus laurentii*	HM469461	Mbingo(NW) Mbalgong(CE)	99%	4
*Cyberlindnera saturnus*	KJ706378	Mbingo(NW) Bambui(NW) Njinikejum(NW) Makepe(LI)	99%	15
*Cyberlindnera subsufficiens*	KY103114	Mbingo(NW) Bambui(NW) Njinikejum(NW) Makepe(LI) Babanki(NW) Eloundem(CE)	99%	28
*Debaryomyces nepalensis*	KP132012	Makepe(LI)	100%	1
*Torulaspora globosa*	KY105654	Mbingo(NW) Bambui(NW) Makepe(LI) Babanki(NW) Eloundem(CE)	99%	21
*Torulaspora delbrueckii*	KP132792.1	Mbingo(NW)	99%	1
*Saccharomyces cerevisiae*	KY105016	Babanki(NW) Eloundem(CE)	99%	2
SP.NOV.1	LC229712	Njinikejum(NW)	98%	1
SP.NOV.2	FM178396	Bambui(NW)	90%	1
SP.NOV.3	KY101957.1	Bambui(NW)	87%	1
SP.NOV.4	HM044867	Njinikejum(NW)	99%	1
SP.NOV.5	KY102188	Bambui(NW)	96%	3
SP.NOV.6.	KU218502	Babanki(NW) Eloundem(CE)	95%	2

**Table 3:** The distribution of yeast species in nine locations within Cameroon. Numbers in table represent the number of isolates in each species (and the percentage of each species) in each location. Our analysis rejected the null hypothesis of no difference in yeast species distribution among the geographic locations (P < 0.001).

Species	Northwest				Littoral	Centre			
	Mbingo	Bambui	Njinikejum	Babanki	Makepe	Eloundem	Mbalgong	Mbandoumou	Simbock
*C. boidinii*	0(0)	2(14)	0(0.0)	0(0)	0(0)	0(0)	0(0)	0(0)	0(0)
*C. pseudolambica*	2(7)	0(0)	3(16)	1(9)	1(4)	0(0)	0(0)	0(0)	0(0)
*C. tropicalis*	0(0)	2(14)	12(63)	0(0)	6(24)	0(0)	0(0)	0(0)	0(0)
*C. laurentii*	3(10)	0(0)	0(0)	0(0)	0(0)	0(0)	1(100)	0(0)	0(0)
*C. saturnus*	6(21)	3(21)	1(5)	0(0)	5(20)	0(0)	0(0)	0(0)	0(0)
*C. subsufficiens*	10(34)	1(7)	1(5)	2(18)	6(24)	8(73)	0(0)	0(0)	0(0)
*D. nepalensis*	0(0)	0(0)	0(0)	0(0)	1(4)	0(0)	0(0)	0(0)	0(0)
*T. globosa*	7(24)	1(7)	0(0)	6(55)	6(24)	1(9)	0(0)	0(0)	0(0)
*T. delbrueckii*	1(3)	0(0)	0(0)	0(0)	0(0)	0(0)	0(0)	0(0)	0(0)
*S. cerevisiae*	0(0)	0(0)	0(0)	0(0)	0(0)	1(9)	0(0)	0(0)	0(0)
*SP.NOV.1	0(0)	0(0)	1(5)	0(0)	0(0)	0(0)	0(0)	0(0)	0(0)
*SP.NOV.2	0(0)	1(7)	0(0)	0(0)	0(0)	0(0)	0(0)	0(0)	0(0)
*SP.NOV.3	0(0)	1(7)	0(0)	0(0)	0(0)	0(0)	0(0)	0(0)	0(0)
*SP.NOV.4	0(0)	0(0)	1(5)	1(9)	0(0)	0(0)	0(0)	0(0)	0(0)
*SP.NOV.5	0(0)	3(21)	0(0)	0(0)	0(0)	0(0)	0(0)	0(0)	0(0)
*SP.NOV.6.	0(0)	0(0)	0(0)	1(9)	0(0)	1(9)	0(0)	0(0)	0(0)
Total number	29	14	19	11	25	11	1	0	0

### Yeast diversity and distribution

The yeast isolation rates differed significantly between different geographical regions. The four sampled sites in the Northwest Region yielded the most yeast isolates: At Mbingo, 29 of the 51 soil samples contained yeasts (56.8%), with the most commonly found yeast species being *C. subsufficiens* (34.4%), followed by *T. globosa* (24.13%), *C. saturnus* (20.6%), *C. laurentii* (10.3%), *C. pseudolambica* (6.8%), and *T. delbrueckii* (3.4%). At Njinikejum, we obtained yeasts from 19 of the 50 soil samples (38%). At this site, *C. tropicalis* was the most prevalent (63.1%), followed by three isolates of *C. pseudolambica* (15.7%), and one isolate (5.2%) each of *C. subsufficiens, C. saturnus*, SP.NOV.1, and SP.NOV.4. At Bambui, we obtained yeasts from 14 of the 50 soil samples (28%), with species distribution as follows: three isolates (21.4%) each of *C. saturnus* and SP.NOV.5; two isolates (14.2%) each of *C. tropicalis* and *C. boidinii*, and one isolate (7.1%) each of *C. subsufficiens, T. globosa*, SP.NOV.2, and SP.NOV.3. At Babanki, eleven yeast strains were obtained from 49 soil samples (22%), including six isolates of *T. globosa* (54.5%), two isolates of *C. subsufficiens* (18.1%), and one isolate (9.0%) each of *C. pseudolambica*, SP.NOV.4, and SP. NOV.6 (Tables 2 and 3).

Compared to the Northwest Region, the yeast isolation rate was lower for soil samples from the Centre Region, with only 12 of the 246 (8.05%) soil samples found to contain yeasts: At Eloundem of the Centre Region, we obtained yeasts from 11 of the 98 soil samples (11.2%). These included eight isolates of *C. subsufficiens* (72.7%), and one isolate (9.0%) each of *T. globosa, S. cerevisiae*, and SP. NOV. 6. At Mbalgong of the Centre Region, only one yeast isolate (*Cryptococcus laurentii*) was obtained from the 51 soil samples (1.9%). Overall, the rate of yeast isolation from soil samples originating from the Northwest Region was statistically higher than that from the Centre Region (P = < 0.00001). The Littoral Region was excluded from the statistical comparison due to the fact that only one location was sampled in this region.

Among the obtained yeasts, two species, *C. subsufficiens* and *T. globose*, were commonly found among the geographic samples from both the Northwest and the Central Regions, corresponding to 25.4% and 19% of all the yeasts respectively in our total yeast population. In contrast, several species (SP.NOV.1, SP.NOV.2, SP.NOV.3, SP.NOV.4, SP.NOV.5, *C. boidinii, T. delbrueckii*) were each isolated from only one location from the Northwest Region, while *D. nepalensis* was isolated only from the Littoral Region (Makepe).

### ITS phylogeny construction

Figure 1 depicts the phylogenetic relationships constructed based on the ITS sequences of the 110 yeast isolates as well as their closely related GenBank sequences. Bootstrap support for individual branches was obtained using 1000 random replications (Fig. 1). The analyses showed close relationships between the two species in the genus *Cyberlindnera, C. saturnus*, and *C. subsufficiens*.

### PCR fingerprinting results

Based on the combined PCR fingerprinting results of the M13 and (GACA)_4_ primers, we constructed a dendrogram among isolates to illustrate the genetic diversity within individual species in our samples. Below we briefly describe the results, with each species separated into a different paragraph.

In total, twenty PCR fingerprint genotypes were found among the 21 *T. globosa* isolates. The only shared genotype was between two isolates from Babanki of the Northwest Region. Of the remaining 19 isolates/genotypes; seven were from Mbingo (NW), six in Makepe (LI), five from Babanki (NW), and one each from Eloundem (CE) and Bambui (NW). Though some isolates from the same local or regional populations were more similar to each other, isolates from the three regions did not exclusively cluster based on their geographic locations but were dispersed among each other (Supplementary Fig. S1). This result is consistent with frequent gene flow among the local yeast populations in Cameroon.

Similar to that in *T. globosa*, a high genotypic diversity was observed in the *C. tropicalis* population. Specifically, each of the 20 isolates of *C. tropicalis* belonged to a different genotype. The 20 isolates were from two regions: the Northwest region and the Littoral region. The majority of the isolates/genotypes (12 of the 20) came from Njinikejum (NW), six from Makepe (LI), and the remaining two from Bambui (NW). The isolates from Njinikejum and Makepe were inter-dispersed with each other on the phylogram, consistent with some degree of gene flow among the regions for C. *tropicalis* (see Fig. S2).

The twenty-eight isolates of *C. subsufficiens* belonged to 25 PCR fingerprinting genotypes (Fig. S3). Thirteen of the 25 genotypes were from the Northwest, including nine genotypes from Mbingo, two from Babanki, and one each from Bambui and Njinikejum. Six of the 25 genotypes were from Makepe (LI) and eight were from Eloundem (CE). Four isolates from Makepe (LI) and Mbingo (NW) shared two genotypes, with each genotype shared by one isolate from each of the two locations. The lack of geography -based clustering again suggest gene flow among local and regional populations of *C. subsufficiens* in Cameroon.

The fifteen isolates of *C. saturnus* belonged to 15 different genotypes (Fig. S4). Six of the genotypes were found in Mbingo (NW), three from Bambui (NW), five from Makepe (LI), and the remaining one was from Njinikejum (NW). There was no shared genotype within or among the different locations for this species. However, isolates from the NW and LI were interspersed among each other in the dendrogram (Fig. S4), consistent with gene flow between the two regions for this species.

The seven isolates of *C. pseudolambica* belonged to six genotypes (Fig. S5). Six of the seven isolates were from the Northwest with each of the six having a different genotype, including two genotypes from Mbingo (NW) and three genotypes from Njinikejum (NW). The only isolates from outside of the NW region [i.e., from Makepe (LI)] shared the genotype with one isolate from Babanki (NW).

The four isolates of *C. laurentii* belonged to three genotypes (Fig. S6). Of the three genotypes, two were from Mbingo (NW), with one genotype containing two isolates from this region. The remaining genotype was represented by one isolate from Mbalgong.

Of the remaining 10 (putative) species, each was represented by three or fewer isolates, and our PCR fingerprinting results showed that they all had different PCR fingerprinting genotypes. For species represented by only one isolate each, their Simpson’s genotype diversity index was not calculated (Table 4).

**Table 4:** Strain genotype, sample size, and the genetic diversity of common yeast species in each region in Cameroon.

Species	No. isolates	No. genotypes	Genotype diversity	Geographic location (Region) #n of isolates)
*Candida boidinii*	2	2	1	Bambui (NW) #2
*Candida pseudolambica*	7	6	0.9	Mbingo (NW) #2 Njinikejum (NW) #3 Makepe (LI) #1 Babanki (NW) #1
*Candida tropicalis*	20	20	1	Bambui (NW) #2 Njinikejum (NW) #12 Makepe (LI) #6
*Cryptococcus laurentii*	4	3	0.8	Mbingo (NW) #3 Mbalgong (CE) #1
*Cyberlindnera saturnus*	15	15	1	Mbingo (NW) #6 Bambui (NW) #3 Njinikejum (NW) #1 Makepe (LI) #5
*Cyberlindnera subsufficiens*	28	27	0.9	Mbingo (NW) #9 Bambui (NW) #2 Njinikejum (NW) #1 Makepe (LI) #6 Babanki (NW) #2 Eloundem (CE) #8
*Torulaspora globosa*	21	20	0.9	Mbingo (NW) #7 Bambui (NW) #1 Makepe (LI) #6 Babanki (NW) #6 Eloundem (CE) #1
*SP.NOV.5*	3	3	1	Bambui (NW) #3
*SP.NOV.6*	2	2	1	Babanki (NW) #1 Eloundem (CE) #1

### Yeast species diversity based on geographical sites

The yeast species richness and Simpson’s species diversity index for different sites within each of the three regions in Cameroon were calculated. The highest yeast species richness was found in Bambui (NW): eight species were found among the 14 yeast isolates with a species diversity index of 0.89. Three local populations from Mbingo, Makepe, and Njinikejum contained six species each, from among 29 (diversity = 0.78), 25 (diversity = 0.81), and 19 (diversity = 0.58) isolates, respectively. Of the remaining locations, five species were found from the 11 isolates in Babanki, (diversity = 0.7); four species among 11 isolates in Eloundem (diversity = 0.48), and only one isolate (species) in Mbalgong.

### Relationship between yeast species diversity and other factors

The pH values in our soil samples ranged from pH 6.4 to 7.10, with the highest value found in Mbandoumou. The sites of the Centre Region generally had lower pH values than other sites. However, we found no apparent correlation between soil pH and yeast biodiversity (Pearson correlation coefficient = −0.18, p < 0.6). Interestingly, however, when we compared soil yeast isolation rates with those of *Aspergillus fumigatus* from the same soil samples, a statistically significant negative correlation was found (Pearson correlation coefficient = −0.67, p < 0.04). Specifically, the presence of *A. fumigatus* in the soil was more likely to be associated with no yeast than those without *A. fumigatus* (see Figs S7 and S8).

## Discussion

One hundred and ten yeast isolates belonging to six genera and 16 (putative) species were isolated from 493 soil samples. Table 2 provides a list of isolated species (15 ascomycetes and one basidiomycete) and their rates in Cameroonian regions/sites representing two climatic zones (Tropical Monsoon and Tropical Savanna climates). Our study yielded several yeast species that have been frequently reported from soil samples in other parts of the world. These included *C. tropicalis*, *C. saturnus, C. subsufficiens*, and *T. globosa*^22–25^. These four species were found at more than one site in our samples (Fig. 1 and Table 2) and they represented about 25% of the total yeast isolates obtained in our soil samples. In addition, PCR fingerprinting analyses showed that, within each of these four species, some genotypes were shared between regions/sites. In addition, isolates were not clustered exclusively based on geographic locations. Our results from the analyses of multiple yeast species indicate a consistent pattern of underlying gene flow between sites and regions within the Cameroonian soil yeast populations. Below we focus on the relevance of the four common yeast species in discussing the implications of the results.

*Cyberlindnera subsufficiens* and *C. saturnus* are two of the 22 yeast species in the genus *Cyberlindnera*^26^. Species in this genus are commonly found in soils and plant surfaces collected in several countries/regions such as the Himalayas, Austria, and Liberia^27^. Interestingly, isolates of *C. saturnus* as well as several other yeast species from natural environments have been reported to be capable of producing killer toxins (mycocines) with a wide spectrum of activities against competing fungal (including yeast) species^22,28,29^. It’s unknown at present whether the isolates of *C. saturnus* and *C. subsufficiens* obtained here from Cameroon can also produce toxins and kill other fungi. However, based on the significant negative correlation between the isolation rates of yeasts and the filamentous fungus *A. fumigatus* described recently in the same Cameroonian soils, it is possible that there are antagonistic interactions between yeasts and *A. fumigatus* in their natural environments. Further studies are needed in order to identify the nature of this potential antagonistic interaction and their potential application for biocontrol of fungal infectious diseases. Aside from fungal toxins, the isolates obtained here may have other applied importance. For example, a recent study reported that a strain of *C. saturnus* from a tropical mangrove wetland in India showed the ability to assimilate xylose and produce xylitol, an industrially important compound^30^. The large number of strains of *C. saturnus* as well as other yeasts isolated here represent a potential resource from which to screen for industrially, medically, and agriculturally important activities.

Given the monsoon and tropical climates in our study sites, the high rate of isolation of *C. tropicalis* was not surprising. In our study, *C. tropicalis* was the most prevalent in soil samples from Njinikejum in the Northwest Region (63.1%, Table 2). Our finding may have significant medical implications for this region. *Candida tropicalis* is among the most common opportunistic yeast pathogens in humans and is especially prevalent in tropical regions, responsible for up to 66% of cases of candidemia^24,31^. A recent meta-analysis on candida species responsible for oral candidiasis in HIV and AIDS–infected African patients revealed that *C. tropicalis* was the second most common yeast species causing invasive fungal infections, accounting for 22% of all cases in immunocompromised patients^32^. Furthermore, a recent study showed that *C. tropicalis* might be prone to become resistant to a variety of antifungal drugs^33^. Our results suggest that local public health in Cameroon should be aware of the epidemiology of fungal infections in this region.

The fourth species we would like to discuss here is *T. globosa*. Similar to the other three species described above, *T. globosa* is broadly distributed in the soil environments, especially in tropical regions. In our samples, *T. globosa* represents 19.1% of the 73 yeasts isolated from the Northwest (Tropical Monsoon Climate) and one of the 12 yeasts from the Tropical Savanna Climate (Table 2). Several studies have reported positive effects of strains of *T. globosa* in the biocontrol of phytopathogens. For example, *T. globosa* was reported to be effective at controlling the sugarcane fungal pathogen *Colletotrichum sublineolum*^25^. Its abundance in the rhizosphere as reported previously is also consistent with its ecological roles in the soil ecosystems^34,35^.

Of the 110 yeast isolates obtained from the Cameroon soil samples, nine had ITS sequences that exhibited significant sequence divergence with all the deposited sequences in the current NCBI GenBank database and the UNITE database, greater than those that have been shown for their closely related species pairs. These nine isolates belong to six groups within which the strains shared >97% ITS sequence identity among each other. Of these six groups, four showed ITS sequence identity of less than 97% to any known sequence while two showed 98% and 99% of sequence identities to two known sequences. However, there was no formal species name attached to these two sequences. Based on the most commonly used criteria for operational taxonomic unit (OTU) identification of fungi, these six groups likely represent six novel yeast species. The closest relatives of these six species were distributed in several genera including *Cyberlindnera* (closest species were *C. subsufficiens* and *C. saturnus*), *Hanseniaspora* (closest species was *H. occidentalis)*, *Candida* (closest species included *C. orthopsilosis*, *C. maltosa*, *C. berkhoutiea*), and *Saccharomyces* (Fig. 1). The findings in the present study are consistent with significant novel species diversity revealed in an earlier study for soil samples from Africa based on metagenome analyses^36^.

We noticed that there was very limited sequence difference at the ITS locus between *C. subsufficiens* and *C. saturnus*. Indeed, only one nucleotide site showed a difference between these two species at this locus: at site 173 of the internal transcribed spacer 1 region with *C. subsufficiens* containing a gap at the site, whereas *C. saturnus* containing *C* at that position. Similar results have also been reported in many other yeast species pairs at the ITS region^11,16^. Despite the low level of sequence divergence between the two species, these two species can be distinguished using the diagnostic nucleotide at this particular position. However, the difference is too small to meet the general barcode gap requirement. Indeed, sequences at secondary or even tertiary barcodes are needed for further confirmation^11,37^.

The two main geographic regions sampled here, the Northwest and the Centre Regions, showed different yeast isolation rates and yeast species distributions. At present, the reasons for the observed differences are not known. One potential factor is temperature differences among the regions. Among these three regions, the Northwest Region has an average June temperature of about 20 °C (range 18–23 °C), the Littoral Region about 26 °C (range of 23–29 °C), and the Centre Region about 25 °C (range 23–30 °C). Previous studies have found that ambient temperature and the availability of water and organic substances favor the growth of fungi, including yeasts. In this study, we found that among the three temperatures (25 °C, 30 °C, and 37 °C) that we tested, all the 110 yeast isolates grew best at 30 °C, followed by 25 °C, with 37 °C supporting the least growth (data not shown). In addition, for the soil samples that did not show any yeast in the first round of isolation at 30 °C, additional attempts were made at 25 °C, 30 °C, and 37 °C. However, there was no new yeast from those soil samples that didn’t show any in the initial screening at 30 °C. Thus, given that we obtained high yeast isolation rates from the Northwest and the Littoral Regions but low rate at the Centre Region and that all yeasts grew best at 30 °C, the natural temperature differences among the regions, as well as the incubation temperatures that we used at 25 °C, 30 °C and 37 °C for isolating yeasts in the lab, are unlikely major contributors to the observed differences in yeast isolation rates among the three regions.

Another potential reason for the different yeast isolation rates among regions may be related to differences in soil factors such as soil pH, nutrient levels, and other soil microorganisms. Earlier studies demonstrated that acidic pH favors the growth of fungi, including yeasts, while alkaline pH favors other microorganisms such as bacteria^38^. In the present study, however, there was no observed correlation between yeast colonization and soil pH (p = 0.6). On the other hand, we found a statistically significant negative correlation between *A. fumigatus* presence and yeast presence in these soil samples, suggesting that competition among fungi might be an important factor determining yeast distribution. Furthermore, an additional study also reported the influence of vegetation properties and soil type on soil yeast diversity^39^.

We would like to note that, for two main reasons, the observed isolation rates and species richness of culturable yeasts likely under-estimated the true native yeast colonization rates and yeast diversity in Cameroonian agricultural soils. First, though we obtained multiple (~50) samples from each of the nine locations, we only obtained 1 gram of soil for each sample. The use of more soil per sample may increase the chance of recovering yeasts from each sample. However, more soil would also likely result in multiple yeast species on the same plate. Because it’s difficult to distinguish yeast species based on colony morphology, choosing one colony from multiple on the same plate but representing different yeast species would create biases for downstream analyses. Thus, the weight of 1 gram per sample (and 0.1–0.2 gram per analysis) was chosen in our study to minimize the probability of multiple yeast species within each soil sample while allowing us to enrich any live yeast cells that might be present in each soil sample. A previous study suggested one gram of typical soil may contain up to 1000 culturable yeast cells^40^. If any live yeast cell exists in a soil sample, we believe our protocol should lead to its enrichment and successful isolation. Regardless, increasing the amount of soil per sample should result in more microbes (including yeasts) sampled and isolated. Second, we only used the generic yeast growth and isolation media SD broth and YEPG agar. While all known culturable yeast species can grow on these media, it’s possible that there are unknown or novel yeast species that are unable to grow in/on these two media. To further explore the entire spectrum of yeast species diversity in our soil samples, a culture-independent approach would be needed. However, because yeasts are phylogenetically broadly distributed in fungi, there is currently no primer/probe that specifically target all yeasts but no filamentous fungi. As a result, the soil yeast diversity study using the culture-independent approach must be part of the broader fungal metagenomic study. Even with this approach, unknown or novel yeasts would likely be missed due to the lack of reference sequence information in current databases for those yeasts to compare with. It should also be noted that such a culture-independent approach would only yield information about yeast species distribution and that the patterns of intra-specific genetic variations within individual yeast species could not be analyzed. Combining a culture-independent metagenomic approach with the culture-based approach will allow scientists to obtain a more realistic understanding of both fungal (including yeast) species diversity and intra-specific genetic variation within individual culturable yeasts.

A high genotypic diversity was found for all yeast species recovered from the Cameroon soil samples. This result is consistent with the hypothesis that these species have likely existed in Cameroon for a long time and are likely native residents of the soil environments there. Furthermore, the lack of obvious genotype clustering according to their geographic locations suggest evidence of gene flow among the locations for multiple species. Both natural forces such as wind and water as well as human activities such as the exchange of agricultural and commercial goods could have contributed to some of the mixed clustering of genotypes.

In summary, we obtained 110 yeast isolates from 493 soil samples from Cameroon. These yeasts were identified as belonging to 16 (putative) species, with ten having already been described previously and the remaining six likely representing new species. Eight of 16 species were present in more than one location. Soil samples from different regions in Cameroon had different yeast isolation rates ranging between 0%-56% and that yeast species distributions were not random across the regions. At present, the reasons for the observed differences in yeast isolation rates remain largely unknown. Importantly, strains from the same species were genetically very diverse, with evidence of gene flow among sites and regions in Cameroon for multiple yeast species. Some of these yeasts are relevant to human health and others may be of economic importance in industry and in agriculture as biocontrol agents against plant fungal pathogens. Despite the high percentage of potentially novel yeast species and the high genotypic diversity within individual species that we observed here, we believe that additional diversities must exist in Cameroonian agricultural soils. The application of culture-independent methods should help us uncover those novel diversities.

### Statements on study approvals

We confirm that all methods in this study were carried out in accordance with relevant guidelines and regulations at McMaster University. In addition, all experimental protocols were approved by the President’s Biosafety Advisory Committee at McMaster University.

## Electronic supplementary material

Supplementary Information

## Data Availability

All data described in the study are presented in the manuscript. The ITS sequences for the 110 isolates have been deposited into the GenBank under the following accession numbers MG817527-MG817636.
