# Supplementary material for: Diversity and relationships among strains of culturable yeasts in agricultural soils in Cameroon
Source: Sci Rep. 2018 Oct 24;8:15687. doi: 10.1038/s41598-018-34122-2 (PMC6200750; doi:10.1038/s41598-018-34122-2)
Supplement: Supplementary file 1 — Supplementary Information [file 41598_2018_34122_MOESM1_ESM.docx]

**Diversity and relationships among strains of culturable yeasts in agricultural soils in Cameroon**

Renad Aljohani, Himeshi Samarasinghe, Tabi Ashu, Jianping Xu*

Department of Biology, McMaster University, 1280 Main St West, Hamilton, Ontario, L8S 4K1, Canada

Corresponding Author: [jpxu@mcmaster.ca](mailto:jpxu@mcmaster.ca)

Running Title: Yeast diversity in Cameroon

Key Words: Yeast, ITS sequencing, PCR-fingerprinting, Geography, Candida, Cyberlindnera

**Supplementary figures**

Figure S1: Genotype diversity based on PCR-fingerprinting of *Torulaspora globosa*


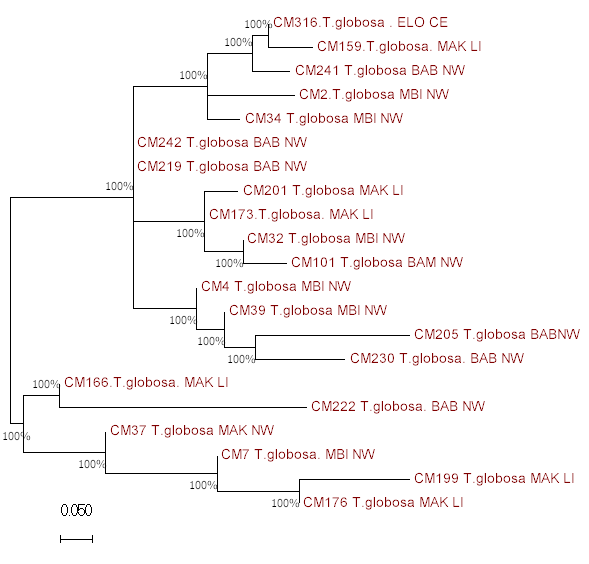


Figure S2: Genotype diversity based on PCR-fingerprinting of *Candida tropicalis*


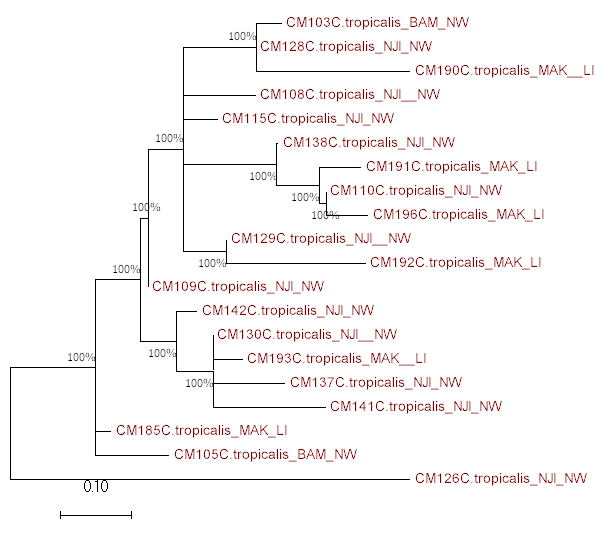


Figure S3: Genotype diversity based on PCR-fingerprinting of *Cyberlindnera subsufficiens*


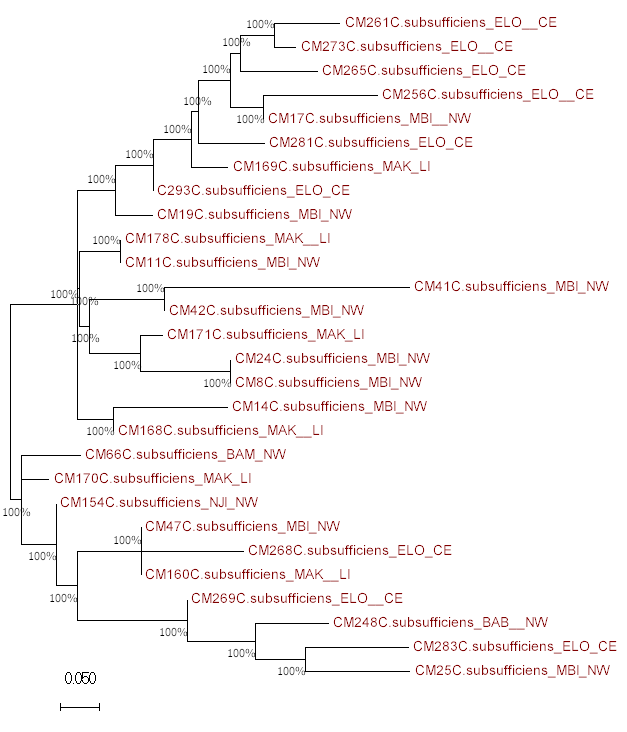


Figure S4: Genotype diversity based on PCR-fingerprinting of *Cyberlindnera saturnus*


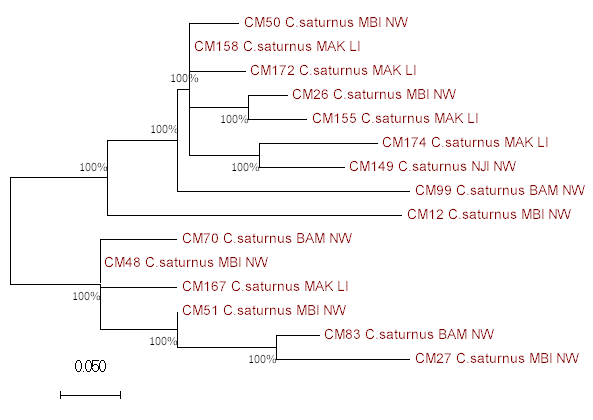


Figure S5: Genotype diversity based on PCR-fingerprinting of *Candida pseudolambica*


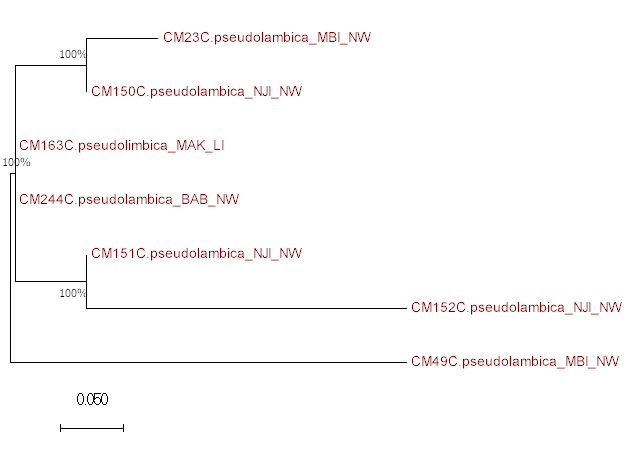


Figure S6: Genotype diversity based on PCR-fingerprinting of *Cryptococcus laurentii*

*
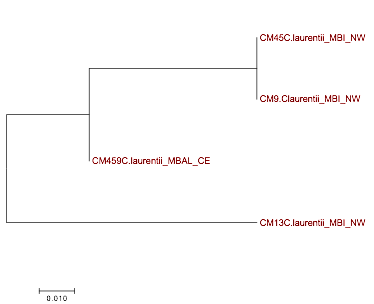
*

Figure S7: The relationship between yeast isolation rate and Aspergillus fumigatus isolation rate of the same soil samples

Figure S8 Correlation between the isolation Rate of yeast and Aspergillus Fumigatus.
